# A strategy for constructing aneuploid yeast strains by transient nondisjunction of a target chromosome

**DOI:** 10.1186/1471-2156-10-36

**Published:** 2009-07-13

**Authors:** Kirk R Anders, Julie R Kudrna, Kirstie E Keller, BreAnna Kinghorn, Elizabeth M Miller, Daniel Pauw, Anders T Peck, Christopher E Shellooe, Isaac JT Strong

**Affiliations:** 1 Biology Department, Gonzaga University, 502 E Boone Avenue, Spokane, WA 99258, USA

## Abstract

**Background:**

Most methods for constructing aneuploid yeast strains that have gained a specific chromosome rely on spontaneous failures of cell division fidelity. In *Saccharomyces cerevisiae*, extra chromosomes can be obtained when errors in meiosis or mitosis lead to nondisjunction, or when nuclear breakdown occurs in heterokaryons. We describe a strategy for constructing N+1 disomes that does not require such spontaneous failures. The method combines two well-characterized genetic tools: a conditional centromere that transiently blocks disjunction of one specific chromosome, and a duplication marker assay that identifies disomes among daughter cells. To test the strategy, we targeted chromosomes III, IV, and VI for duplication.

**Results:**

The centromere of each chromosome was replaced by a centromere that can be blocked by growth in galactose, and *ura3::HIS3*, a duplication marker. Transient exposure to galactose induced the appearance of colonies carrying duplicated markers for chromosomes III or IV, but not VI. Microarray-based comparative genomic hybridization (CGH) confirmed that disomic strains carrying extra chromosome III or IV were generated. Chromosome VI contains several genes that are known to be deleterious when overexpressed, including the beta-tubulin gene *TUB2*. To test whether a tubulin stoichiometry imbalance is necessary for the apparent lethality caused by an extra chromosome VI, we supplied the parent strain with extra copies of the alpha-tubulin gene *TUB1*, then induced nondisjunction. Galactose-dependent chromosome VI disomes were produced, as revealed by CGH. Some chromosome VI disomes also carried extra, unselected copies of additional chromosomes.

**Conclusion:**

This method causes efficient nondisjunction of a targeted chromosome and allows resulting disomic cells to be identified and maintained. We used the method to test the role of tubulin imbalance in the apparent lethality of disomic chromosome VI. Our results indicate that a tubulin imbalance is necessary for disomic VI lethality, but it may not be the only dosage-dependent effect.

## Background

Any change in chromosome number through the gain and/or loss of part of a haploid set of chromosomes is known as aneuploidy. Aneuploidy leads to defects in the growth and development of an organism (reviewed in [1,2]). In cases of chromosome gain, the phenotype of an aneuploid is influenced by the effects of two phenomena: (1) a general, physiological response to excess protein expression, leading to a slowing of cell proliferation [3], and (2) protein stoichiometry imbalances specific to genes on the extra chromosome [1-3]. A complete understanding of the complex phenotype caused by any specific aneuploid karyotype requires an ability to manipulate chromosome contents and copy number.

In the yeast *Saccharomyces cerevisiae*, aneuploids have been isolated in a number of ways over the years. Strains with extra chromosomes have arisen spontaneously among lab strains (for examples, see [4,5]), and have been generated through meiosis of triploids [6]. Specific disomes (haploids carrying an extra chromosome, karyotype N+1) have been isolated by differentially marking two homologs in a diploid, then selecting for meiotic segregants that contain both homologs (for example, [7]). An alternative method to generate disomes makes use of transient heterokaryons that form during mating between *kar1*^- ^and *KAR1*^+ ^haploids [8]. At a certain frequency, chromosomes are transferred from one nucleus to another before one nucleus is lost. By differentially marking homologs in the parents and selecting for progeny cells that retain both homologs, haploid progeny carrying a disomic chromosome have been isolated [9,10]. This method, termed chromoduction, was used to select for 14 of the possible 16 disomes of yeast in a recent systematic study of aneuploidy [3]. Although the methods described above are clearly effective at isolating disomic strains of yeast, each of them requires a spontaneous failure of chromosome segregation during cell division. The mechanisms that underlie these failures (the breakdown of nuclear integrity in a cell containing multiple nuclei or the bypass of the spindle assembly checkpoint to allow nondisjunction [11,12]) are not well understood, and may lead to additional, unplanned genetic changes.

We have devised a method for generating disomes that does not rely on spontaneous failure in cell division integrity. Instead, the method specifically blocks mitotic segregation of the target chromosome alone. The method comprises a novel combination of two well-characterized genetic tools, a conditional centromere [13] and a duplication marker [14]. When these are placed at the centromere of a target chromosome, disjunction of the chromosome can be transiently blocked to generate disomic cells, some of which are selectively identified by the duplication marker.

We report the results of a proof-of-concept test with chromosomes III, IV, and VI. The method efficiently generated disomic III and IV strains, but did not produce disomic VI unless *TUB1 *copy number was also increased.

## Results and discussion

### General strategy to induce and select for a duplicated chromosome

**Figure 1:**
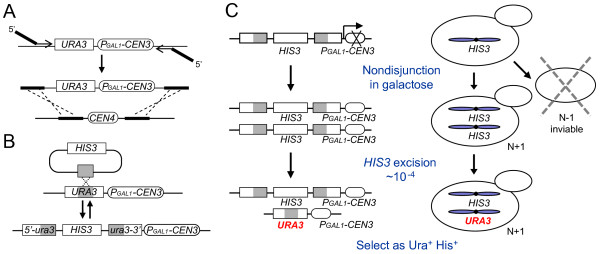
**Strategy for modifying and duplicating target chromosomes**. (A) PCR amplifies P_GAL1_-*CEN3 URA3 *from the plasmid pGALCEN-JC3-13 [13] and, upon transformation into yeast, replaces the target centromere by homologous recombination. (B) The *HIS3 *plasmid pKA52 is integrated into *URA3 *adjacent to the conditional centromere, disrupting the *URA3 *open reading frame and generating direct repeats (shaded). At an approximate frequency of 10^-4^, *HIS3 *is lost by homologous recombination between the direct repeats, regenerating a functional *URA3 *gene (reverse arrow). The recipient strain carries the deletion alleles *ura3*Δ*0 *and *his3*-Δ*200*. (C) A haploid carrying a modified chromosome from (B) is grown in galactose for one cell division, generating N+1 and N-1 cells by nondisjunction. Since the *ura3::HIS3 *marker is present in two copies, cells with *URA3 *and *HIS3 *can be produced by *HIS3 *excision and are identified as Ura^+^His^+ ^papillae on selective medium.

The strategy involves modifying the centromeric region of a target chromosome so that (1) the centromere can be inactivated temporarily to cause nondisjunction and (2) daughter cells that obtained two copies of the target chromosome can be selected. The chromosome modification strategy is outlined in Figure 1A and 1B. The conditional centromere construct P_GAL1_-*CEN3 URA3 *[13] is PCR-amplified with primers that provide homology to sequence flanking the target centromere. The PCR fragment is transformed into yeast and integrated into the target site by homologous recombination, replacing the endogenous centromere [15] (Figure 1A). P_GAL1_-*CEN3 *functions as an autonomous centromere when placed into plasmids or chromosomes, and its function can be blocked when galactose induces *GAL1 *promoter activity [13,16]. In galactose, many kinetochore proteins do not assemble on the centromere, but within 20 minutes of a switch to glucose, kinetochore assembly is observed [17]. Once the conditional centromere is in place, a marker that can detect changes in ploidy is generated at *URA3*, following a strategy devised by Chan and Botstein [14]. A plasmid containing *HIS3 *and an internal fragment of *ura3 *is transformed into yeast, integrating into *URA3 *and disrupting its function (Figure 1B, forward arrow). Chromosome duplication is detected based on the properties of this integrated plasmid. The integration creates a 390 bp direct repeat of a portion of *ura3*. Homologous recombination between the repeats causes excision and loss of *HIS3 *and regeneration of functional *URA3 *(Figure 1B, reverse arrow). (For simplicity, we refer to this event as excision of *HIS3 *even though it can occur by a variety of homologous recombination events including excision, gene conversion and unequal sister chromatid exchange [18].) Because *HIS3 *can be lost and *URA3 *regenerated, the locus exists in one of two mutually exclusive states: either it will contain an intact *HIS3 *or an intact *URA3*, but not both. Duplication of this marker locus, followed by excision of *HIS3*, should lead to cells that contain both *URA3 *and *HIS3*. Such cells should exhibit Ura^+^His^+ ^phenotypes.

The strategy for inducing and selecting disomic cells is outlined in Figure 1C. A haploid strain with a chromosome containing the conditional centromere and duplication marker is exposed to galactose during one cell division cycle. The conditional centromere is inactivated and the target chromosome fails to disjoin during mitosis. Among the daughter cells that contain two copies of the target chromosome, a fraction will spontaneously excise one of the *HIS3 *markers to generate *URA3*. These cells will be recovered as Ura^+^His^+ ^colonies on selective medium.

### Construction of modified chromosomes as targets for duplication

As a proof-of-concept test, we chose three chromosomes (III, IV, and VI) to modify and target for duplication. Chromosomes III and VI are among the smallest yeast chromosomes (317 and 270 kb, respectively), whereas chromosome IV is the one of the largest (1.53 Mb) [19]. We anticipated that chromosomes IV and VI would pose a challenging test of this method because they were among the least frequently isolated disomes using chromoduction [10], and disomic strains containing a single extra chromosome VI failed to be isolated by Torres et al. [3]. Rather, colonies selected for chromosome VI disomy also contained extra, unselected chromosomes, suggesting that simple chromosome VI disomes may be inviable [3]. We constructed each modified chromosome twice, independently, and tested for concordance of phenotype. The chromosomes were constructed in diploids (or in haploids that were subsequently mated to wild type) to produce cells heterozygous for the conditional centromere and duplication markers. When the heterozygotes were sporulated, the modified chromosomes segregated 2:2 and produced haploid colonies on rich medium that were indistinguishable from wild-type segregants (Additional file 1A). We conclude that the centromeric modifications themselves do not lead to growth phenotypes.

To test whether the conditional centromeres in our strains could be inhibited by galactose to cause nondisjunction, we tested for chromosome loss by constructing diploid strains that were heterozygous for a conditional centromere marked with *URA3*. Growth in galactose, followed by plating to glucose-containing medium, resulted in the appearance of many Ura^- ^colonies. Consistent with Hill and Bloom's observations [13], galactose exposure for 1–2 generations led to the loss of the *URA3 *marker in approximately 50% of the cells (Additional file 1B). In the case of diploids carrying modified chromosome IV, most of the galactose-induced Ura^- ^colonies exhibited a severe, slow-growth phenotype, suggesting that the Ura^- ^colonies were the result of losing the target chromosome (Additional file 1C). We conclude that the conditional centromere allows for galactose-inducible nondisjunction in our strains.

**Figure 2:**
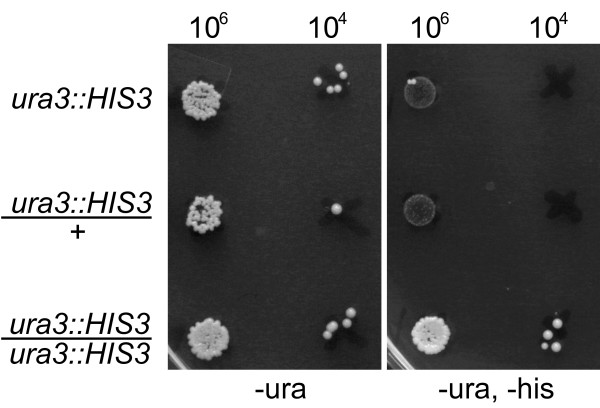
**Papillation pattern in strains that contain one or two copies of the duplication marker *ura3::HIS3***. Ura^-^His^+ ^strains were grown overnight in YPD rich medium. 10^6 ^and 10^4 ^cells from each culture were spotted to plates containing selective media lacking uracil (-ura) or uracil and histidine (-ura, -his). Papillae were scored after 3 days. The strains shown are KAY579 (haploid), KAY626 (hemizygous diploid), and KAY625 (homozygous diploid).

To characterize the *ura3::HIS3 *duplication marker, we measured the frequency of *HIS3 *excision and reconstitution of *URA3 *at each modified centromere. Each marker excised *HIS3 *to produce Ura^+ ^papillae at a frequency near 10^-4 ^(Table 1). Galactose did not alter this frequency. When cultures were placed under selection for Ura^+^His^+ ^papillae, strains with two copies of the marker produced colonies at the frequency of *HIS3 *excision, whereas strains with one copy produced colonies at a much lower frequency (Figure 2). To produce these rare Ura^+^His^+ ^papillae, the strains with a single marker had to undergo spontaneous duplication of the chromosome (or the marker itself) in addition to *HIS3 *excision. Under selection for this kind of duplication marker, Chan and Botstein found that most events (85%) were likely catastrophic increases in ploidy rather than single chromosome gains [14]. We conclude that the *ura3::HIS3 *marker constructed here should identify cells that have gained an extra copy of the modified chromosome.

**Table 1:** Frequency of Ura^+ ^cells in haploid strains containing *ura3::HIS3 *integrated next to a centromere

**Chromosome containing *ura3::HIS3***	**Galactose exposure prior to selection**	**Frequency of Ura^+^**
*III*	-	3.4 ± 3.2 × 10^-4^
*IV*^*a*^	-	1.0 ± 0.0 × 10^-4^
*VI*^*b*^	-	9.7 ± 1.8 × 10^-5^
*VI*^*b*^	+	1.3 ± 0.6 × 10^-4^

Haploid cells were grown overnight in YPD rich medium. 10^6 ^cells were plated onto selective medium lacking uracil. Colonies were counted after 3 days. The mean frequency and standard deviation obtained from at least 4 independent cultures are shown.

^*a *^Only 2 independent cultures were tested.

^*b *^Cultures were grown in supplemented minimal medium containing raffinose. Where indicated, galactose was added to 1.5% and culture was grown for 1.3 doublings prior to plating onto selective medium. Diluted cultures were also plated to YPD to score viable cell density. Ura^+ ^frequency was computed per viable cell plated. A paired t-test indicates that the frequencies were not different (2-tailed, P > 0.1).

### Targeted chromosomes are duplicated after nondisjunction is induced

**Figure 3:**
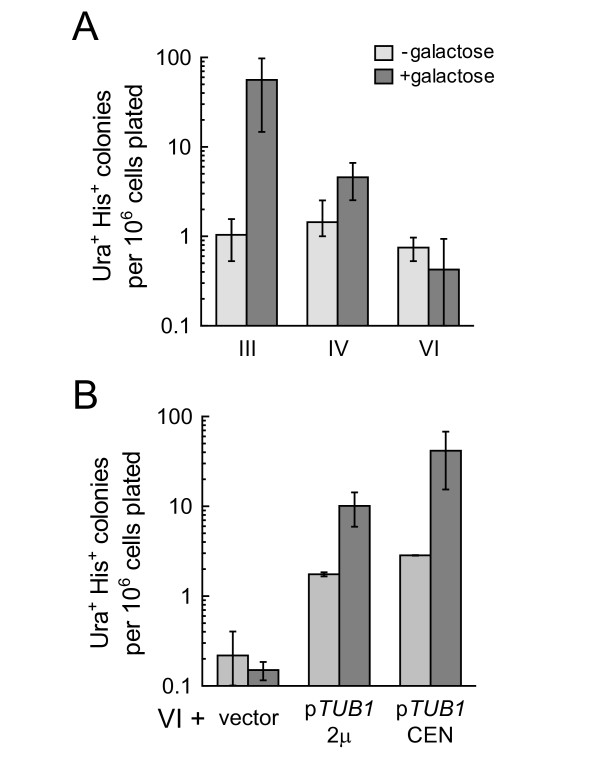
**Frequency of appearance of Ura^+^His^+ ^papillae in strains carrying a modified chromosome**. Haploid strains were grown to log phase in raffinose-containing medium, exposed to galactose (dark bars) or not (light bars), then plated to glucose-containing medium lacking uracil and histidine. Papillae were scored after 3 days. Bars represent the mean frequencies ± standard deviations from at least 2 independent trials. (A) Strains carrying modified chromosome III were KAY418 and KAY419; modified IV, KAY614 and KAY619; modified VI, KAY539 and KAY568. (B) Strains carrying modified chromosome VI were KAY591 and KAY628 that harboured vectors pRS425 (2-micron) or pRS315 (CEN), or *TUB1 *plasmids pRB327 (2-micron) or pKA55 (CEN). The frequencies in strains treated with no galactose (light bars) are not statistically different from each other, with one exception. Strains with modified VI that carry p*TUB1 *plasmids exhibited higher spontaneous frequencies than did VI strains without p*TUB1 *(Tukey-Kramer test, p < 0.05).

If nondisjunction of the conditional centromere causes chromosome gain as well as chromosome loss, induced disomic cells should be detectable by an increase in the frequency of appearance of Ura^+^His^+ ^colonies. To select directly for Ura^+^His^+ ^disomes, haploid cells carrying a modified chromosome were grown to log phase in medium containing raffinose, the cultures were split, and galactose was added to one of the resulting cultures. After 1–1.3 culture doublings, cells were spread to selective plates. In the absence of galactose, all strains produced spontaneous Ura^+^His^+ ^papillae at a frequency of approximately 10^-6 ^(Figure 3). For strains containing modified chromosome III or IV, growth in galactose increased the frequency of Ura^+^His^+ ^papillae formation (Figure 3A), suggesting that many of the papillae had developed from disomes that formed by galactose-induced nondisjunction. In contrast, the strains containing modified chromosome VI showed no increase in the appearance of Ura^+^His^+ ^papillae when grown in galactose. (We consider this lack of chromosome VI duplication below.)

**Figure 4:**
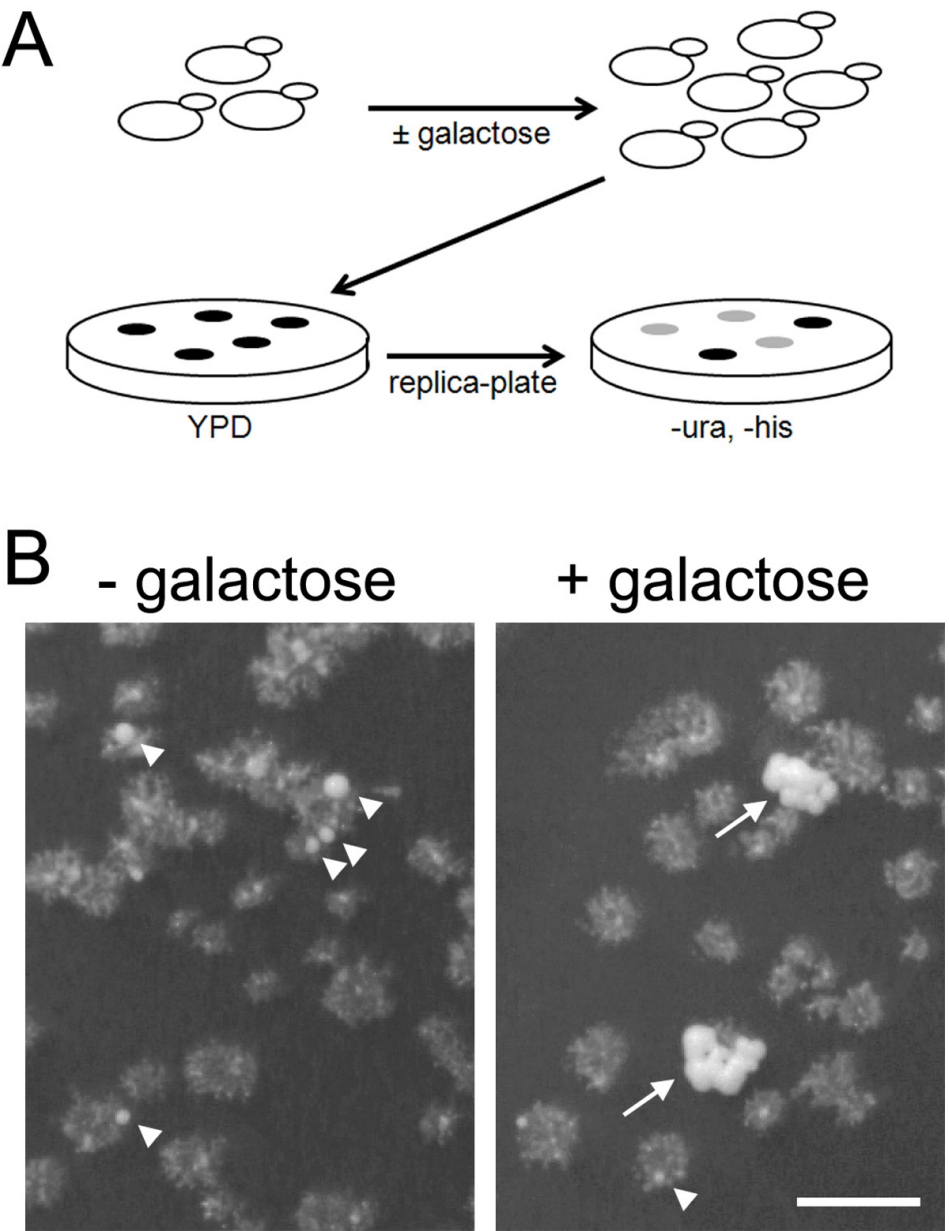
**Delayed selection strategy to identify chromosome IV disomes**. (A) Cells were grown approximately one doubling in the presence of galactose to induce nondisjunction, diluted and plated to YPD rich medium to allow all viable cells to form colonies. If any colonies are clones of stable disomes, they should produce Ura^+^His^+ ^papillae at high frequency when replica-plated to selective medium. Spontaneous duplications that occur after colony formation on YPD should appear as isolated papillae on selective medium. (B) Photographs of replica plates after 3 days. Medium lacks uracil and histidine. Triangles indicate isolated papillae appearing on non-growing "ghost colonies." Arrows indicate single colonies on which numerous papillae appear. Strain shown is KAY614. Bar is 5 mm.

In addition to direct selection for Ura^+^His^+ ^colonies, we tested whether a "delayed selection" scheme could identify candidate disomes (Figure 4A). Cells carrying a modified chromosome IV were grown in galactose for 1.3 culture doublings, diluted, and plated to rich medium. The resulting colonies were replica-plated to selective medium and screened for colonies that produce numerous Ura^+^His^+ ^papillae (Figure 4B). Among colonies from 7 independent galactose-treated cultures, 4.8% behaved as candidate disomes, whereas only 0.37% of the colonies from cultures that were not exposed to galactose looked like candidate disomes (standard deviations were 2.8% and 0.43%, respectively).

**Figure 5:**
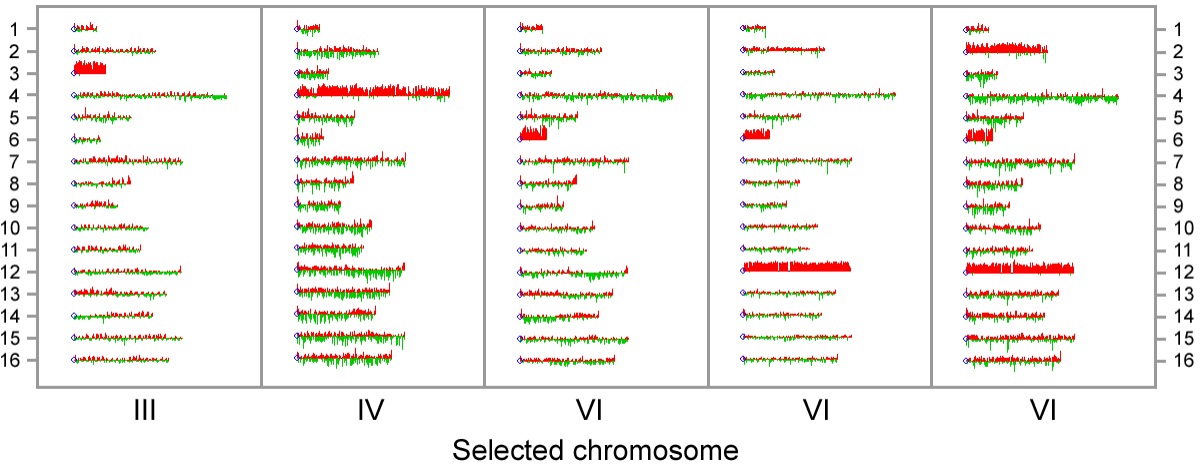
**Karyotypes of Ura^+^His^+ ^candidate disomes**. Haploid strains carrying a modified chromosome were treated with galactose and plated to select Ura^+^His^+ ^papillae as described in the text. To assess chromosome copy number, DNA from Ura^+^His^+ ^isolates (red) was combined with DNA from a haploid parent strain (green) and hybridized to microarrays containing the genomic collection of yeast open reading frames. Log-transformed red:green ratios are displayed in histogram format for each gene along each chromosome, using the Karyoscope viewer of Java Treeview [44]. Results from representative arrays are shown (disomic III: KAY495; disomic IV: KAY638; disomic VI: KAY605; disomic VI, XII: KAY679; disomic II, VI, XII: KAY681). Complete data from all arrays are deposited at GEO [45].

To determine whether Ura^+^His^+ ^isolates were disomic, we examined chromosome copy number by microarray-based comparative genomic hybridization (array CGH). Among galactose-induced cultures, we tested 3 isolates that targeted chromosome III and 6 that targeted IV (1 selected directly and 5 from the delayed selection protocol). All of these isolates contained a single, extra copy of the target chromosome (see Figure 5 for examples of each karyotype). In contrast, among spontaneous Ura^+^His^+ ^isolates, 1/6 that targeted chromosome IV (1/3 direct selection, 0/3 delayed selection) displayed disomy. We conclude that this method, placing a conditional centromere and duplication marker on a target chromosome, allows for efficient isolation of newly formed disomic strains of yeast.

### Extra copies of *TUB1 *allow the isolation of chromosome VI disomes

Since galactose exposure did not increase the frequency of Ura^+^His^+ ^papillae in strains carrying a modified chromosome VI (Figure 3A), we did not have confidence that the selected colonies were disomic. Indeed, when 5 isolates were examined by array CGH, aneuploidy was not detected (data not shown). This result is consistent with the notion that chromosome VI disomy is lethal, as suggested by previous studies that found VI disomy to be either rare or absent [3,6,10,20]. For example, disomic haploids are frequently found among the rare, viable spores produced by interspecific hybrids of *Saccharomyces cerevisiae *and *paradoxus *[20]. Hunter et al. examined 300 such spores by CHEF gel analysis and observed no occurrences of chromosome VI disomy among the nine chromosomes detectable by this method, consistent with a possible lethality of disomic chromosome VI [20]. Similarly, in a systematic study of chromoduction, Dutcher found that when chromosome VI disomy was selected, the frequency of its appearance was much lower than other chromosomes of its size, perhaps because there is strong selection against chromosome VI disomes [10]. More recently, Torres et al. used chromoduction to construct and study a nearly complete set of N+1 disomic strains [3]. However, when chromosome VI disomy was selected and the resulting cells were examined by array CGH, the unselected chromosomes I and XIII were also present. The absence of single chromosome VI disomes suggests that such a karyotype may be lethal, and that the presence of chromosomes I and XIII suppresses this lethality [1,3].

If chromosome VI disomy is lethal, expression of one or more genes on the extra chromosome VI may cause stoichiometry imbalances severe enough to prevent viability. There are several well-studied genes on chromosome VI that are known to be deleterious upon overexpression, including *CDC14*, *ACT1 *and *TUB2 *[21-25]. For example, *TUB2*, which codes for beta-tubulin, has been shown to be exquisitely dosage-sensitive. Overexpression of *TUB2 *causes lethality, even when a single, extra copy is integrated into a haploid genome [23]. This lethality can be suppressed by increased expression of alpha-tubulin in the cell, encoded by the chromosome XIII genes *TUB1 *and *TUB3 *[26,27]. The observations of Torres et al. support the idea that the extra dose of *TUB2 *contributes to the lethality of disomic chromosome VI: in the viable strains that are disomic for I, VI, and XIII, the extra copy of chromosome XIII can supply the cell with additional alpha-tubulin, eliminating the stoichiometry imbalance caused by the extra copy of *TUB2 *[3].

Clearly, a second copy of *TUB2 *in a haploid is sufficient to cause lethality, as demonstrated by the *TUB2 *integration experiments of Katz et al. [23]. But when the entire chromosome VI is duplicated, is the duplication of *TUB2 *necessary for the extra copy of chromosome VI to cause lethality? If it is, then eliminating the tubulin imbalance alone should allow for the viability of chromosome VI disomes. However, if additional chromosome VI genes cause dosage imbalances severe enough to prevent viability, then simply eliminating the tubulin imbalance should not suppress the lethality of disomic chromosome VI.

We used the chromosome duplication method described here to test whether the tubulin imbalance is necessary for the lethality of VI disomy. We supplied *TUB1*-containing plasmids to haploid strains carrying modified chromosome VI, then induced nondisjunction and selected for duplication of the *ura3::HIS3 *marker. In contrast to strains without excess *TUB1*, many Ura^+^His^+ ^candidate disomes did appear after exposure to galactose when the cells harboured a *TUB1 *plasmid (Figure 3B). The effect occurred when *TUB1 *was carried on a low-copy *CEN *plasmid or on a high-copy 2-micron plasmid.

Most of the Ura^+^His^+ ^isolates, although viable, produced slow-growing, tiny colonies (Additional file 2). We grew 9 isolates in liquid culture, extracted DNA and performed array CGH. Although one culture did not exhibit aneuploidy, the other 8 were disomic for chromosome VI: 3 isolates were simple disomes, 3 isolates also contained an extra, unselected chromosome XII, and 2 contained extra chromosomes II and XII (see Figure 5 for examples of each karyotype). Since a number of colonies were isolated that contained the single, extra chromosome VI, and since these were only isolated when extra copies of *TUB1 *were present, we conclude that minimizing (or eliminating) the effect of *TUB2 *overexpression allows for the viability of chromosome VI disomes. *TUB2 *overexpression is therefore essential for the inviability of chromosome VI disomy.

Although viable, the chromosome VI disomes exhibited growth defects (Additional file 2). We do not know whether these defects are the result of residual tubulin dosage problems, other gene-specific effects, or a combination of the numerous ways that aneuploidy affects phenotype [1]. Further, it is not clear what role, if any, the unselected chromosomes II and XII play. Since the aim of this report is to describe our method for manipulating chromosome copy number, a complete study of the basis for chromosome VI dosage phenotypes will be reported elsewhere.

## Conclusion

We have described a new method for inducing and selecting disomic yeast strains which does not rely on spontaneous errors in chromosome segregation. The method allowed for efficient isolation of disomic strains carrying either chromosome III or IV. We used the method to test a specific dosage relationship between chromosome VI and the alpha-tubulin gene *TUB1*, and found that chromosome VI disomes could be isolated with this method when plasmid-borne *TUB1 *was present. Our proof-of-concept test was therefore successful for each of the three chromosomes we tested.

In principle, any strain that already contains a conditional centromere could be supplemented with a duplication marker and used to generate disomic aneuploids. Reid et al., for example, have generated conditional centromeres on all 16 chromosomes for inducing loss of heterozygosity [28,29], and these chromosomes could be further modified as described above for the induction and selection of disomes. The method should be useful in studies investigating the genetic basis of aneuploid phenotypes, and any study that wishes to efficiently duplicate a chromosome *de novo*.

## Methods

### Media and genetic manipulations

Standard methods were used for growth and genetic analysis of yeast [30], except that YPD medium was supplemented with 50 mg/l adenine sulfate and 20 mg/l uracil. Sporulation was induced as described [31]. Unless otherwise noted, carbon sources were supplemented to 2% (wt/vol) and cells were grown at 30°C in supplemented minimal media to maintain plasmids or unstable integrations. Cell density was determined using a hemacytometer.

### Construction of plasmids

Plasmids are listed in Table  2[13,26,32,33]. Oligonucleotides were designed with Primer3 [34] and are listed in Additional file 3. Standard methods of DNA manipulation were used, unless otherwise noted [30,35]. To construct the plasmid pKA52, a 390 bp fragment internal to *URA3 *was amplified from pGALCEN-JC3-13 template DNA with the use of oligonucleotide primers URA3int_F and URA3int_R in a high-fidelity *Pfu *polymerase chain reaction (PCR) (Stratagene). This fragment was digested with *Bam*HI and *Eco*RI, then ligated into the *Bam*HI/*Eco*RI sites of pRS303. The plasmid pKA55 was constructed using homologous recombination in yeast [36] to replace the *URA3 *marker of pRB326 with *LEU2*. A 3.7 kb *Sph*I/*Pvu*II fragment containing *LEU2 *and flanking vector sequence was cut from pRB327, gel-purified with the Qiaquick gel extraction kit (Qiagen), then combined with *Sma*I-linearized pRB326 DNA to co-transform a *leu2*^- ^yeast strain. pKA55 was recovered by isolating DNA from Leu^+ ^transformants and transforming *E. coli *strain JM109. To confirm that pKA55 contained functional *TUB1*, it was transformed into a *tub1*Δ strain containing the *TUB1-URA3 *plasmid pRB326. Cells were grown in medium supplemented with uracil to allow loss of pRB326, plated to YPD, then replica-plated to medium selecting for Leu^+^. Colonies were identified that were Leu^+^Ura^-^, indicating that the sole source of *TUB1 *was from pKA55.

**Table 2:** Plasmids used in this study

**Plasmid**	**Genotype**	**Source**
pGALCEN-JC3-13	P_GAL1_-*CEN3, URA3*	[13]
pRS303	*HIS3*	[32]
pRS315	*LEU2, CEN6, ARSH4*	[32]
pRS425	*LEU2, 2 μ*	[33]
pRB326	*TUB1, URA3, CEN4, ARS1*	[26]
pRB327	*TUB1, LEU2, 2 μ*	[26]
pKA52	*HIS3, ura3 *(390 bp fragment)	This study
pKA55	*TUB1, LEU2, CEN4, ARS1*	This study

### Construction of yeast strains

Yeast strains used in this study are listed in Table 3. All strains were derived from the S288C-related BY4741 and FY strains [37,38]. KAY519, KAY530 and KAY587 were descended from a cross between BY4741 and the FY3-derived DBY10147.

**Table 3:** Yeast strains used in this study

**Strain**	**Genotype**	**Source**
BY4741	*MAT***a ***his3Δ1 leu2Δ0 met15Δ0 ura3Δ0*	[38]
DBY10147	*MATα*	D. Botstein (Princeton University)
DBY8869	*MAT***a ***his3-Δ200 ura3-52*	D. Botstein (Princeton University)
DBY8871	*MATα his3-Δ200 ura3-52*	D. Botstein (Princeton University)
DBY8923	*MAT***a ***his3-Δ200 ura3-52 ade2Δ leu2-Δ1 lys2Δ*	D. Botstein (Princeton University)
DBY8925	*MATα his3-Δ200 ura3-52 ade2Δ leu2-Δ1 lys2Δ*	D. Botstein (Princeton University)
KAY519	*MAT***a ***leu2Δ0*	This study
KAY530	*MATα his3-Δ200 ura3Δ0*	This study
KAY587	*MATα his3-Δ200 leu2Δ0 ura3Δ0*	This study
KAY418, 419	*MAT***a ***his3-Δ200 ura3-52 cen3::*P_GAL1_-*CEN3 ura3::HIS3(at CEN3)*	This study
KAY541, 542	*MAT***a ***his3-Δ200 ura3Δ0 cen3::*P_GAL1_-*CEN3 ura3::HIS3(at CEN3)*	This study
KAY614, 619	*MAT***a ***his3-Δ200 ura3Δ0 cen4::*P_GAL1_-*CEN3 ura3::HIS3(at CEN4)*	This study
KAY539, 568	*MAT***a ***his3-Δ200 ura3Δ0 cen6::*P_GAL1_-*CEN3 ura3::HIS3(at CEN6)*	This study
KAY591, 628	*MAT***a ***his3-Δ200 leu2Δ0 ura3Δ0 cen6::*P_GAL1_-*CEN3 ura3::HIS3(at CEN6)*	This study
KAY579	*MATα his3-Δ200 ura3Δ0 ade2Δ cen6::*P_GAL1_-*CEN3 ura3::HIS3(at CEN6)*	This study
KAY626	*MATα*/*MAT***a ***his3-Δ200/his3-Δ200 LEU2/leu2Δ0 ura3Δ0/ura3Δ0 ade2Δ/ADE2 cen6::*P_GAL1_-*CEN3/CEN6 ura3::HIS3(at CEN6)*	This study
KAY625	*MATα*/*MAT***a ***his3-Δ200/his3-Δ200 LEU2/leu2Δ0 ura3Δ0/ura3Δ0 ade2Δ/ADE2 cen6::*P_GAL1_-*CEN3/cen6::*P_GAL1_-*CEN3 ura3::HIS3(at CEN6)/ura3::HIS3(at CEN6)*	This study

Haploid yeast strains were constructed to contain a modified chromosome (III, IV, or VI) that harbors a conditional centromere and a set of duplication markers (Figures 1A, B and Additional file 4). To construct KAY418 and KAY419, which carry a modified chromosome III, DNA containing P_GAL1_-*CEN3 URA3 *from plasmid pGALCEN-JC3-13 was transformed into strains DBY8923 and DBY8869, respectively, replacing chromosomal *CEN3 *as described [13]. To generate the *ura3::HIS3 *duplication marker, these strains were transformed with the integrative plasmid pKA52, which had been cut at the unique *Stu*I site within its *URA3 *fragment. The strains were then backcrossed. Genetic linkage to chromosome III markers and centromere confirmed the *CEN3 *location of the integrated DNA. To generate KAY541 and KAY542, the strains were crossed to KAY530 to replace *ura3-52 *with *ura3Δ0 *as described below.

To construct KAY614 and KAY619, which carry a modified chromosome IV, a 2.6 kb fragment containing P_GAL1_-*CEN3 URA3 *was amplified with high-fidelity *Phusion *PCR (Finnzymes) using the primers CEN4_REPL_F and CEN4_REPL_R and pGALCEN-JC3-13 template DNA. Each primer contains at its 5' end a 50 nt sequence identical to that found adjacent to *CEN4*. The PCR fragment was gel-purified (Qiaquick gel extraction kit, Qiagen) and used to transform the diploid strain DBY8869 × DBY8925. An independently-amplified fragment was used to transform the isogenic diploid DBY8871 × DBY8923. Each resulting strain was transformed with *Stu*I-digested pKA52 to generate *ura3::HIS3*. To confirm that the conditional centromere integrated at *CEN4*, DNA from Ura^-^His^+ ^transformants was amplified by PCR using the primers CEN4_F (located outside the integration site near *CEN4*) and URA3_int_R (located in the conditional centromere sequence). To generate haploid strains, the heterozygous, transformed diploids were sporulated and the *ura3::HIS3 *marker segregated 2:2. When these strains were used to select for duplication of chromosome IV (see Induction and selection of N+1 disomes, below), some Ura^+^His^+ ^derivatives were found in which the *ura3-52 *allele on chromosome V (which consists of the full-length *URA3 *gene with a Ty1 insertion [39]) had recombined with *ura3::HIS3 *on chromosome IV to generate *URA3*^+ ^without duplicating the intact target chromosome (data not shown). To prevent this unwanted event, *ura3-52 *was replaced with *ura3Δ0 *by crossing the haploid strains containing P_GAL1_-*CEN3 ura3::HIS3 *to KAY530. Spore clones that contained *ura3Δ0 *were identified by the PCR-amplification of a 550 bp fragment from spore clone DNA using the primers URA3_del_F and URA3_del_R, which flank the *URA3 *coding region.

KAY539 and KAY568, which carry a modified chromosome VI, were constructed with the same methods as were KAY614 and KAY619, except the primers CEN6_REPL_F and CEN6_REPL_R were used to target P_GAL1_-*CEN3 URA3 *to replace *CEN6*, and the primer CEN6_F was used with URA3_int_R in a PCR to confirm that the conditional centromere had integrated at *CEN6*. KAY591 and KAY628 were constructed by crossing KAY539 and KAY568, respectively, to KAY519.

### Induction and selection of N+1 disomes

Haploid strains carrying P_GAL1_-*CEN3 ura3::HIS3 *at the centromere of the target chromosome were grown to saturation (2 days) in supplemented minimal medium that contained raffinose as its nonrepressing carbon source [40], diluted at least 2000-fold into fresh medium and grown overnight to obtain log-phase cultures. The cultures were split when the density was 0.5–1 × 10^7 ^cells/ml. To one half, galactose was added to a final concentration of 1.5%. Cell density was monitored until cultures had grown approximately 1.3 doublings (2.5-fold increase in density). The cells were pelleted, resuspended in water, diluted and plated to YPD and selective media lacking uracil or lacking both histidine and uracil. Ura^+ ^colonies were scored to determine *HIS3 *excision frequency. Ura^+^His^+ ^colonies were scored to determine frequency of duplication and excision, and were picked as candidate disomes (direct selection method). Colonies that grew on YPD were scored to determine viable cell density of plated cultures, and replica-plated to selective plates lacking uracil (to monitor excision) and to plates lacking histidine and uracil (to monitor duplication and excision). Replica-plated colonies on which many Ura^+^His^+ ^papillae grew were considered candidate disomes and Ura^+^His^+ ^papillae were picked (delayed selection method).

### Microarray-based comparative genomic hybridization (array CGH)

Microarrays were produced by spotting PCR-amplified DNA fragments from approximately 6200 yeast open reading frames (kindly donated by D. Botstein, Princeton University) onto poly-lysine coated glass slides as described [41]. Genomic DNA was isolated by glass bead lysis according to the protocol of Hoffman and Winston [42]. To label each sample, 2 μg DNA was digested with *Hae*III, purified, then resuspended in water. The DNA was boiled in the presence of 15 μg random nonamer nucleotide primers, then cooled on ice. The hybridized primers were extended with the use of 20 units of *exo*^- ^Klenow polymerase (New England Biolabs) in a 50 μl reaction containing 180 μM each of dATP, dGTP, dCTP, 72 μM dTTP, 108 μM 5-(3-aminoallyl)-dUTP, 50 mM NaCl, 10 mM Tris (pH 7.9), 10 mM MgCl_2_, and 1 mM dithiothreitol. After 2 hours at 37°C, EDTA (pH 8.0) was added to 45 mM. The primer-extension products were purified through a DNA Clean and Concentrator-5 spin column (Zymo Research) and resuspended in 50 mM sodium bicarbonate (pH 9). Reactive Cy3 (or Cy5) mono NHS ester dyes (GE Healthcare, Cat. No. PN5661) were coupled to the aminoallyl groups in the DNA as directed by the supplier. The labeled DNA was purified through another spin column and resuspended in 20 μl 10 mM Tris (pH 8.5). Cy3- and Cy5-labeled DNAs were combined and hybridized to the microarrays at 65°C for 18 hours in a solution of 3× SSC, 730 μg/ml PolyA RNA, 240 μg/ml tRNA, 24 mM HEPES buffer (pH 7), and 0.24% SDS. Arrays were washed in 0.05× SSC at room temperature and imaged with a GenePix 4000B scanner (Molecular Devices). Array images were analyzed with ScanAlyze [43]. Data was filtered for signal intensity at least 2-fold above background in both channels, ratios were normalized to average 1 across all unaffected chromosomes, and log ratios were visualized with the Karyoscope viewer of Java Treeview [44].

### Accession number

The raw microarray data, accession number GSE14377, are deposited at GEO [45].

## List of abbreviations

CGH: comparative genomic hybridization; PCR: polymerase chain reaction; YPD: yeast extract, peptone, and dextrose; YP-galactose: yeast extract, peptone, and galactose

## Authors' contributions

KA conceived and designed the experiments, constructed yeast strains, performed yeast genetics and microarray experiments, and wrote the paper. JK constructed yeast strains, performed yeast genetics and microarray experiments, and drafted an early version of the paper. KK constructed yeast strains. BK and AP constructed a plasmid and yeast strains. EM and CS performed yeast genetics experiments. DP constructed yeast strains and performed yeast genetics experiments. IS constructed a plasmid, and performed yeast genetics and microarray experiments. All authors read and approved the final manuscript.

## Supplementary Material

Additional file 1 **Characterization of conditional centromere**. (A) Conditional centromere does not cause growth defects on glucose. The heterozygous parent of KAY614, containing P_GAL1_-*CEN3 *and *ura3::HIS3 *at the *CEN4 *locus, was sporulated and tetrads were dissected onto YPD rich medium. (B) Kinetics of galactose-induced chromosome loss. A diploid strain, heterozygous for P_GAL1_-*CEN3 URA3 *at *CEN3*, was grown in YPD to log phase, washed and incubated in YP-galactose, plated to YPD, then phenotyped. The non-repressing sugar raffinose was used in later experiments instead of glucose [40], which is expected to allow more rapid induction of *GAL1 *promoter activity. (C) Galactose-induced loss of chromosome IV yields unstable 2N-1 phenotype. A diploid strain, heterozygous for P_GAL1_-*CEN3 URA3 *at *CEN4*, was grown overnight in YPD or YP-galactose, then plated to YPD. Most of the small colonies were Ura^- ^and unstable, rapidly reverting to normal growth but remaining Ura^-^. This is consistent with endoreduplication of the remaining chromosome IV, as observed by Alvaro et al. [29]. Click here for file

Additional file 2 **Galactose induction of candidate chromosome VI disomes**. Galactose induces the appearance of many small Ura^+^His^+ ^papillae in a strain carrying a modified chromosome VI. Strain KAY628, harbouring the *LEU2*-marked *TUB1 *plasmid pKA55, was grown to log phase in raffinose-containing medium, split, and one-half was exposed to galactose for 1.3 culture doublings. 10^7 ^cells were spread to plates selecting for Ura^+^His^+^Leu^+ ^papillae. Plates were incubated 3 days and photographed. Papillae were picked, colony-purified, then cultured for DNA isolation and array CGH as described in the text. Click here for file

Additional file 3 **Oligonucleotides used in this study**. This table contains nucleotide sequences and genome coordinates of the oligonucleotides used for PCR. Click here for file

Additional file 4 **Strain construction summaries**. This file contains flowcharts that summarize the construction of yeast strains in this study. Click here for file

## References

[B1] Torres EM, Williams BR, Amon A (2008). Aneuploidy: cells losing their balance. Genetics.

[B2] Birchler JA, Veitia RA (2007). The gene balance hypothesis: from classical genetics to modern genomics. Plant Cell.

[B3] Torres EM, Sokolsky T, Tucker CM, Chan LY, Boselli M, Dunham MJ, Amon A (2007). Effects of aneuploidy on cellular physiology and cell division in haploid yeast. Science.

[B4] Cox BS, Bevan EA (1962). Aneuploidy in yeast. New Phytologist.

[B5] Hughes TR, Roberts CJ, Dai H, Jones AR, Meyer MR, Slade D, Burchard J, Dow S, Ward TR, Kidd MJ (2000). Widespread aneuploidy revealed by DNA microarray expression profiling. Nat Genet.

[B6] Campbell D, Doctor JS, Feuersanger JH, Doolittle MM (1981). Differential mitotic stability of yeast disomes derived from triploid meiosis. Genetics.

[B7] Shaffer B, Brearley I, Littlewood R, Fink GR (1971). A stable aneuploid of *Saccharomyces cerevisiae*. Genetics.

[B8] Conde J, Fink GR (1976). A mutant of *Saccharomyces cerevisiae *defective for nuclear fusion. Proc Natl Acad Sci USA.

[B9] Nilsson-Tillgren T, Petersen JGL, Holmberg S, Kielland-Brandt MC (1980). Transfer of chromosome III during *kar *mediated cytoduction in yeast. Carlsberg Research Communications.

[B10] Dutcher SK (1981). Internuclear transfer of genetic information in *kar1-1/KAR1 *heterokaryons in *Saccharomyces cerevisiae*. Mol Cell Biol.

[B11] Rieder CL, Maiato H (2004). Stuck in division or passing through: what happens when cells cannot satisfy the spindle assembly checkpoint. Dev Cell.

[B12] Biggins S, Walczak CE (2003). Captivating capture: how microtubules attach to kinetochores. Curr Biol.

[B13] Hill A, Bloom K (1987). Genetic manipulation of centromere function. Mol Cell Biol.

[B14] Chan CS, Botstein D (1993). Isolation and characterization of chromosome-gain and increase-in-ploidy mutants in yeast. Genetics.

[B15] Baudin A, Ozier-Kalogeropoulos O, Denouel A, Lacroute F, Cullin C (1993). A simple and efficient method for direct gene deletion in *Saccharomyces cerevisiae*. Nucleic Acids Res.

[B16] Hill A, Bloom K (1989). Acquisition and processing of a conditional dicentric chromosome in *Saccharomyces cerevisiae*. Mol Cell Biol.

[B17] Collins KA, Castillo AR, Tatsutani SY, Biggins S (2005). De novo kinetochore assembly requires the centromeric histone H3 variant. Mol Biol Cell.

[B18] Schiestl RH, Igarashi S, Hastings PJ (1988). Analysis of the mechanism for reversion of a disrupted gene. Genetics.

[B19] Cherry JM, Ball C, Weng S, Juvik G, Schmidt R, Adler C, Dunn B, Dwight S, Riles L, Mortimer RK (1997). Genetic and physical maps of *Saccharomyces cerevisiae*. Nature.

[B20] Hunter N, Chambers SR, Louis EJ, Borts RH (1996). The mismatch repair system contributes to meiotic sterility in an interspecific yeast hybrid. Embo J.

[B21] Liu H, Krizek J, Bretscher A (1992). Construction of a *GAL1*-regulated yeast cDNA expression library and its application to the identification of genes whose overexpression causes lethality in yeast. Genetics.

[B22] Moriya H, Shimizu-Yoshida Y, Kitano H (2006). In vivo robustness analysis of cell division cycle genes in *Saccharomyces cerevisiae*. PLoS Genet.

[B23] Katz W, Weinstein B, Solomon F (1990). Regulation of tubulin levels and microtubule assembly in *Saccharomyces cerevisiae*: consequences of altered tubulin gene copy number. Mol Cell Biol.

[B24] Vinh DB, Drubin DG (1994). A yeast TCP-1-like protein is required for actin function in vivo. Proc Natl Acad Sci USA.

[B25] Visintin R, Craig K, Hwang ES, Prinz S, Tyers M, Amon A (1998). The phosphatase Cdc14 triggers mitotic exit by reversal of Cdk-dependent phosphorylation. Mol Cell.

[B26] Schatz PJ, Solomon F, Botstein D (1986). Genetically essential and nonessential alpha-tubulin genes specify functionally interchangeable proteins. Mol Cell Biol.

[B27] Weinstein B, Solomon F (1990). Phenotypic consequences of tubulin overproduction in *Saccharomyces cerevisiae*: differences between alpha-tubulin and beta-tubulin. Mol Cell Biol.

[B28] Reid RJ, Sunjevaric I, Voth WP, Ciccone S, Du W, Olsen AE, Stillman DJ, Rothstein R (2008). Chromosome-scale genetic mapping using a set of 16 conditionally stable *Saccharomyces cerevisiae *chromosomes. Genetics.

[B29] Alvaro D, Sunjevaric I, Reid RJ, Lisby M, Stillman DJ, Rothstein R (2006). Systematic hybrid LOH: a new method to reduce false positives and negatives during screening of yeast gene deletion libraries. Yeast.

[B30] Amberg DC, Burke DJ, Strathern JN (2005). Methods in Yeast Genetics: a Cold Spring Harbor Laboratory course manual.

[B31] Kassir Y, Simchen G (1991). Monitoring meiosis and sporulation in *Saccharomyces cerevisiae*. Methods Enzymol.

[B32] Sikorski RS, Hieter P (1989). A system of shuttle vectors and yeast host strains designed for efficient manipulation of DNA in *Saccharomyces cerevisiae*. Genetics.

[B33] Christianson TW, Sikorski RS, Dante M, Shero JH, Hieter P (1992). Multifunctional yeast high-copy-number shuttle vectors. Gene.

[B34] Rozen S, Skaletsky H (2000). Primer3 on the WWW for general users and for biologist programmers. Methods Mol Biol.

[B35] Sambrook J, Russell DW (2001). Molecular Cloning: A Laboratory Manual.

[B36] Ma H, Kunes S, Schatz PJ, Botstein D (1987). Plasmid construction by homologous recombination in yeast. Gene.

[B37] Winston F, Dollard C, Ricupero-Hovasse SL (1995). Construction of a set of convenient *Saccharomyces cerevisiae *strains that are isogenic to S288C. Yeast.

[B38] Brachmann CB, Davies A, Cost GJ, Caputo E, Li J, Hieter P, Boeke JD (1998). Designer deletion strains derived from *Saccharomyces cerevisiae *S288C: a useful set of strains and plasmids for PCR-mediated gene disruption and other applications. Yeast.

[B39] Rose M, Winston F (1984). Identification of a Ty insertion within the coding sequence of the *S. cerevisiae URA3 *gene. Mol Gen Genet.

[B40] Johnston M, Flick JS, Pexton T (1994). Multiple mechanisms provide rapid and stringent glucose repression of *GAL *gene expression in *Saccharomyces cerevisiae*. Mol Cell Biol.

[B41] DeRisi JL, Iyer VR, Brown PO (1997). Exploring the metabolic and genetic control of gene expression on a genomic scale. Science.

[B42] Hoffman CS, Winston F (1987). A ten-minute DNA preparation from yeast efficiently releases autonomous plasmids for transformation of *Escherichia coli*. Gene.

[B43] ScanAlyze. http://rana.lbl.gov/EisenSoftware.htm.

[B44] Saldanha AJ (2004). Java Treeview – extensible visualization of microarray data. Bioinformatics.

[B45] Gene Expression Omnibus. http://www.ncbi.nlm.nih.gov/geo/.

