# Supplementary figures and images for: A strategy for constructing aneuploid yeast strains by transient nondisjunction of a target chromosome
Source: BMC Genet. 2009 Jul 13;10:36. doi: 10.1186/1471-2156-10-36 (PMC2725114; doi:10.1186/1471-2156-10-36)

Additional file 1

**A**

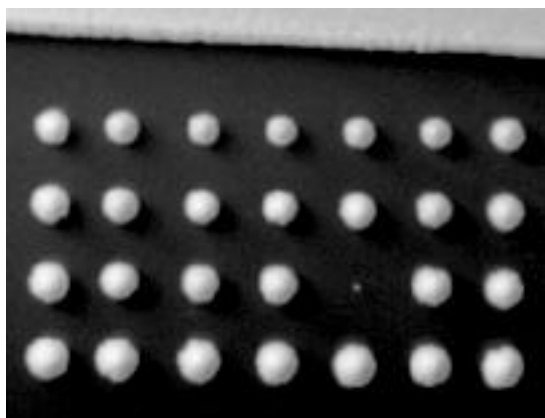

**B**

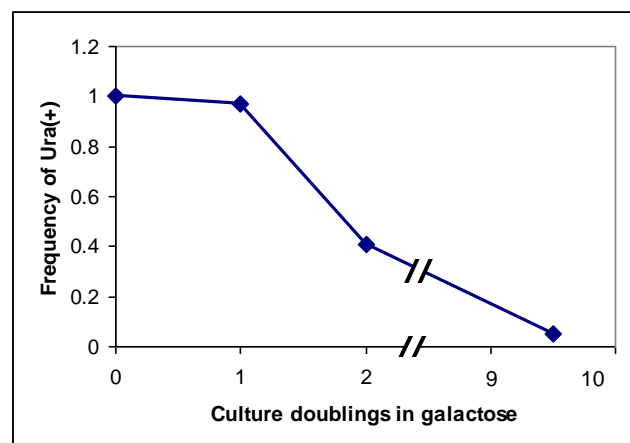

**C**

**Glucose**

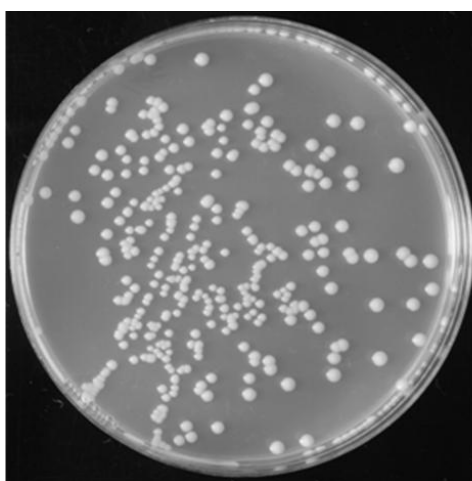

**Galactose**

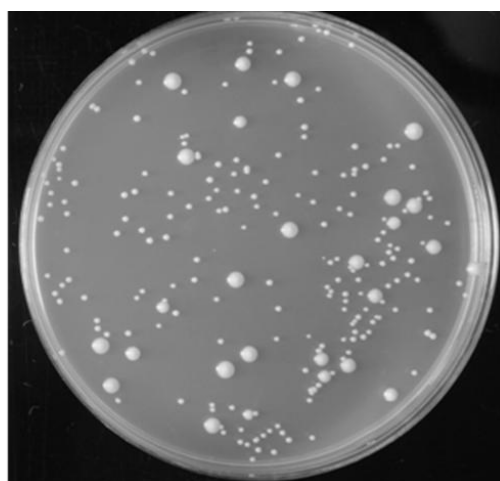

Supplement: Additional file 1 — Characterization of conditional centromere. (A) Conditional centromere does not cause growth defects on glucose. The heterozygous parent of KAY614, containing PGAL1-CEN3 and ura3::HIS3 at the CEN4 locus, was sporulated and tetrads were dissected onto YPD rich medium. (B) Kinetics of galactose-induced chromosome loss. A diploid strain, heterozygous for PGAL1-CEN3 URA3 at CEN3, was grown in YPD to log phase, washed and incubated in YP-galactose, plated to YPD, then phenotyped. The non-repressing sugar raffinose was used in later experiments instead of glucose [40], which is expected to allow more rapid induction of GAL1 promoter activity. (C) Galactose-induced loss of chromosome IV yields unstable 2N-1 phenotype. A diploid strain, heterozygous for PGAL1-CEN3 URA3 at CEN4, was grown overnight in YPD or YP-galactose, then plated to YPD. Most of the small colonies were Ura- and unstable, rapidly reverting to normal growth but remaining Ura-. This is consistent with endoreduplication of the remaining chromosome IV, as observed by Alvaro et al. [29]. [file 1471-2156-10-36-S1.pdf]

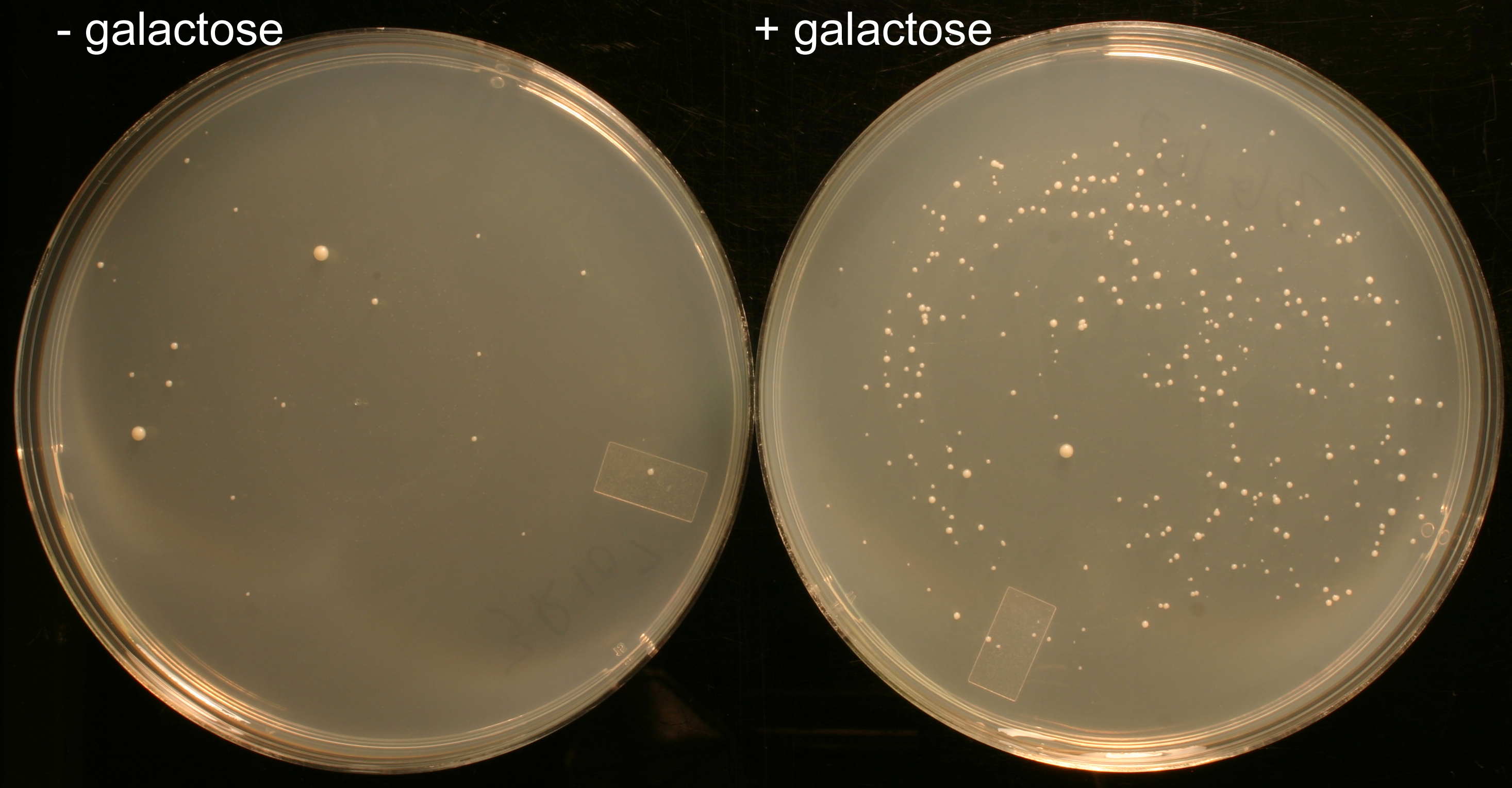

Supplement: Additional file 2 — Galactose induction of candidate chromosome VI disomes. Galactose induces the appearance of many small Ura+His+ papillae in a strain carrying a modified chromosome VI. Strain KAY628, harbouring the LEU2-marked TUB1 plasmid pKA55, was grown to log phase in raffinose-containing medium, split, and one-half was exposed to galactose for 1.3 culture doublings. 107 cells were spread to plates selecting for Ura+His+Leu+ papillae. Plates were incubated 3 days and photographed. Papillae were picked, colony-purified, then cultured for DNA isolation and array CGH as described in the text. [file 1471-2156-10-36-S2.jpeg]
