# Supplementary material for: A strategy for constructing aneuploid yeast strains by transient nondisjunction of a target chromosome
Source: BMC Genet. 2009 Jul 13;10:36. doi: 10.1186/1471-2156-10-36 (PMC2725114; doi:10.1186/1471-2156-10-36)
Supplement: Additional file 3 — Oligonucleotides used in this study. This table contains nucleotide sequences and genome coordinates of the oligonucleotides used for PCR. [file 1471-2156-10-36-S3.pdf]

Additional file 3

Oligonucleotides used in this study

Name	Sequence [a]	Location in genome
URA3_internal_F	ccaggatccGCATTATCCGCCAAGTACAA	V:116401-116420 (*URA3*)
URA3_internal_R	cgcgaattcTGTCATCTAAACCCACACCG	V:116772-116791 (*URA3*)
CEN4_REPL_F	cgactttgtaaaagttcacattagcatacatatattacacgagccagaaCCCCTTCCGCTTATAGTACAG	IV:449607-449655, III:114268-114288. (53 bp to the left of *CEN4*)
CEN4_REPL_R	attttctgtaaagagtttaagctatgaaagcctcggcattttggccgctcCGAGTACAACACCCGATCCT	IV:449870-449920, III:115134-115153. (52 bp to the right of *CEN4*)
CEN6_REPL_F	aaaataatatataaacctgtataatataaccttgaagactatatttcttCCCCTTCCGCTTATAGTACAG	VI:148453-148501, III: 114268-114288. (4 bp to the left of *CEN6*)
CEN6_REPL_R	aatttctaactttaaattttttttgctattataatactaatttctaacttCGAGTACAACACCCGATCCT	VI:148632-148681, III:115134-115153 (10 bp to the right of *CEN6*)
CEN4_F	CACAAATCTGGCTTAATAAAG	IV:449504-449524 (131 bp to the left of the GALCEN integration junction)
CEN4_R	TGACGATAAAACCGGAAGGA	IV:449955-449974 (85 bp to the right of the GALCEN integration junction)
CEN6_F	AGTCGGTACCTATGTGAAACT	VI:148355-148375 (126 bp to the left of the GALCEN integration junction)
CEN6_R	GAAATTACGTACTTTCAACGC	VI:148768-148788 (136 bp to the right of the GALCEN integration junction)
ura3_del_F	TGCGAGGCATATTTATGGTG	V:115867-115886 (300 bp upstream of the *URA3* ORF)
ura3_del_R	GGAGTTCAATGCGTCCATCT	V:117209-117228 (258 bp downstream of the *URA3* ORF)

[a] Lowercase indicates 5’ tail that does not hybridize to original template during amplification. Underlined sequence indicates restriction site. Primers were designed with Primer3 (http://frodo.wi.mit.edu/). 5’ tails were chosen manually.
