# Supplementary material for: A strategy for constructing aneuploid yeast strains by transient nondisjunction of a target chromosome
Source: BMC Genet. 2009 Jul 13;10:36. doi: 10.1186/1471-2156-10-36 (PMC2725114; doi:10.1186/1471-2156-10-36)
Supplement: Additional file 4 — Strain construction summaries. This file contains flowcharts that summarize the construction of yeast strains in this study. [file 1471-2156-10-36-S4.pdf]

Additional file 4

Construction of KAY418 and KAY419, KAY541 and KAY542 (modified III)

haploid: DBY8923 or DBY8969

↓

integrated P_{GAL1}-*CEN URA3* into *CEN3*

↓

integrated pKA52 *HIS3* into *URA3*

↓

backcrossed to wild type, producing KAY418 and KAY419

↓

crossed to *ura3Δ0* to replace *ura3-52*, producing KAY541 and KAY542

Construction of KAY614 and KAY619 (IV) KAY539 and KAY568 (VI)

diploid: DBY8871 x DBY8923 or DBY8925 x DBY8869

↓

integrated PCR-generated P_{GAL1}-*CEN URA3* into *CEN4* or *CEN6*

↓

integrated pKA52 *HIS3* into *URA3*

↓

sporulated, identified segregants carrying P_{GAL1}-*CEN ura3::HIS3*

↓

crossed to *ura3Δ0* to replace *ura3-52*

Construction of KAY591 and KAY628 (modified VI with *leu2Δ0*)

KAY539 or KAY568

↓

crossed to *leu2Δ0*

↓

transformed with *TUB1* plasmid or empty vector
